# The elusive search for a biomarker of dissociative amnesia: a reaction to Dimitrova et al. (2021)

**DOI:** 10.1017/S0033291722001118

**Published:** 2022-10

**Authors:** R. J. C. Huntjens, H. Otgaar, G. H. M. Pijnenborg, I. Wessel

**Affiliations:** 1 Clinical Psychology and Experimental Psychopathology, University of Groningen, Groningen, the Netherlands; 2 Clinical Psychological Science, Maastricht University, Maastricht, the Netherlands; 3 Faculty of Law and Criminology, KU Leuven, Leuven, Belgium; 4 Clinical and Developmental Neuropsychology, University of Groningen, Groningen, the Netherlands

Dimitrova et al. (2021) claimed to have found a biomarker for dissociative amnesia in DID: the volume of the hippocampal subregion CA1. In this commentary, we argue that their claims are overstated.

First, the authors claimed to have investigated the neurobiology of dissociative *amnesia*. However, as an index of dissociative amnesia, they used the *subjective* amnesia scale scores of the DES, a self-report instrument of dissociative symptoms. An *objective* cognitive measure of dissociative amnesia was not included. Additionally, previous studies (i.e. around 15 publications) that did include such an objective measure were not discussed and these studies found (memory) *transfer* of neutral, self-referential/autobiographical, and trauma-related information between identities in DID rather than amnesia (e.g. Huntjens, Verschuere, & McNally, 2012; Marsh et al., 2018).

Second, are their results specific to DID? The authors concluded that ‘the association of CA1 with dissociative amnesia remained significant even after controlling for co-morbidity. This indicates that CA1 volume reduction is primarily driven by dissociative amnesia in DID, not other disorders’ (p. 5). However, this conclusion was based on an analysis controlling for the presence or absence of ‘any’ comorbid disorder. However, when controlling for specific comorbid disorders (e.g. panic disorder, anxiety disorder, see Table S4), a number of correlations between the CA1 volume and reports of amnesia were no longer significant. More importantly, all DID patients had comorbid PTSD. Thus, the results equally favor the interpretation that PTSD and hippocampal CA1 volume reduction are linked, refuting the authors' conclusion of a specific dissociative disorder effect.

A third problematic issue is that their correlational analyses were not corrected for multiple testing. Dimitrova et al., explored associations between 4 dissociation scores and 20 hippocampal indices, yielding 80 correlations. Three were statistically significant (0.030 *p* ⩽ 0.049), pertaining to DES dissociative amnesia and the left and right CA1 regions, and to total DES and Left CA1. These significant effects may well be chance findings and would not have survived correction for multiple testing. Moreover, parts of the dataset already figured in one or more previous publications of the same authors, increasing the number of tested associations even further. For example, Chalavi et al. (2015a, 2015b) investigated an additional 100 correlations between the dissociation / traumatization measures and (other) hippocampal indices. Also, Reinders et al. (2018) investigated associations between dissociation/traumatization measures with different brain areas than the ones in the current paper. Testing so many possible associations in the same data, focusing on only slightly different aspects (e.g. subscales and subareas) in separate publications inflates false-positive rates**.** Furthermore, given the correlational study design, causality cannot be inferred. Observing correlations between specific brain structure volumes and cognitive impairments does not mean that these impairments are *caused* by aberrances in these brain structures. The paper neglects alternative explanations, e.g., the possibility that aberrant metamemory functioning or other factors unrelated to memory underlie smaller hippocampal volume.

Fourth, besides a total score, four subscales were included to index history of abuse: emotional neglect, emotional abuse, physical abuse, and sexual abuse/harassment. All subscales were hypothesized to correlate with global and subfield hippocampal volume. Although only one subscale (emotional neglect) was significantly associated, the authors concluded that ‘traumatization’ had a detrimental effect on hippocampal volume. This is an unbalanced and overgeneralized representation of the findings**.** Moreover, the results in the current paper are inconsistent with previous analyses on (parts of) the same data that showed an absence of significant association between the left and right CA1 region and emotional neglect but correlations with other subscales of this trauma scale (e.g. sexual abuse, physical neglect) and with other subregions of the hippocampus (e.g. left CA2-3, left CA4-DG) (e.g. Chalavi et al., 2015a, 2015b). In sum, a clear conclusion on the association between trauma history and hippocampal (sub)region volume based on these data seems unwarranted.

Finally, the quest to find a biomarker in the form of structural brain damage is incompatible with the mere nature of dissociative amnesia. The DSM-5 emphasizes that dissociative amnesia differs from permanent amnesia that occurs due to neurobiological damage or toxicity preventing memory storage or retrieval. The critical difference is that dissociative amnesia is *always potentially reversible* because the memory has been successfully stored (p. 298). It is precisely for this reason that neuroimaging techniques have been used to *exclude* structural brain damage in the diagnosis of dissociative amnesia (e.g. Brand et al., 2009). The reversible nature of dissociative amnesia is even more evident in patients with DID. Patients with DID report state-dependent changes in conscious access to neutral and trauma-related experiences in different identity states which may fluctuate rapidly in time. This reported controlled reversible state-dependency of dissociative amnesia seems incompatible with a claim of structural brain damage as a biomarker in DID.

In sum, the conclusion that ‘the volume of CA1 can serve as a biomarker for dissociative amnesia’ (p. 5) is overstated and unjustified. Not only has it been argued recently that the conclusion of most published studies seeking biomarkers for behavioral traits might be wrong (Marek et al., 2022), we also question the need for assuming the existence of a special mechanism like dissociation that is supposed to banish traumatic memories from conscious awareness. Alternative explanations for self-reported dissociative amnesia have recently been forwarded. One such explanation suggests that reports of dissociative amnesia may result from *dysfunctional beliefs* fueled by avoidance of the realization of negative events from the past. Examples include beliefs like ‘I would lose control of my life if I allowed myself to remember painful things that happened to me’. An index of these dysfunctional beliefs showed strong associations with reported dissociative symptoms (Huntjens et al., 2022). Conceptualizing reports of dissociative amnesia as the result of dysfunctional beliefs about the self and one's memory functioning may be a way forward out of the controversy surrounding this disorder.
