# The elusive search for a biomarker of dissociative amnesia: A reaction to Dimitrova et al. (2021) – CORRIGENDUM

**DOI:** 10.1017/S0033291722002306

**Published:** 2022-10

**Authors:** R. J. C. Huntjens, H. Otgaar, G. H. M. Pijnenborg, I. Wessel

**Affiliations:** 1 Clinical Psychology and Experimental Psychopathology, University of Groningen, Groningen, the Netherlands; 2 Clinical Psychological Science, Maastricht University, Maastricht, the Netherlands; 3 Faculty of Law and Criminology, KU Leuven, Leuven, Belgium; 4 Clinical and Developmental Neuropsychology, University of Groningen, Groningen, the Netherlands

The authors regret the inclusion of an error in the title of their correspondence.

When originally published, the title for this paper had the year of the article it responds to incorrectly stated as 2022. The correct year for this article should have been 2021.

This has now been corrected.
